# Geriatric trauma hip fractures: is there a difference in outcomes based on fracture patterns?

**DOI:** 10.1186/1749-7922-9-59

**Published:** 2014-12-13

**Authors:** Alicia Mangram, Phillip Moeser, Michael G Corneille, Laura J Prokuski, Nicolas Zhou, Jacqueline Sohn, Shalini Chaliki, Olakunle F Oguntodu, James K Dzandu

**Affiliations:** John C. Lincoln North Mountain Hospital, Phoenix, USA; North Mountain Radiology Group Hospital, Phoenix, USA; Midwestern University – Arizona College of Osteopathic Medicine, Kragujevac, Arizona USA; School of Medicine, University of Missouri – Kansas City, Kansas, USA

**Keywords:** Femoral neck fractures, Hip fractures, Length of stay, Hip fracture patterns, Geriatric G-60

## Abstract

**Background:**

Annually in the US, there are over 300,000 hospital admissions due to hip fractures in geriatric patients. Consequently, there have been several large observational studies, which continue to provide new insights into differences in outcomes among hip fracture patients. However, few hip fracture studies have specifically examined the relationship between hip fracture patterns, sex, and short-term outcomes including hospital length of stay and discharge disposition in geriatric trauma patients.

**Methods:**

We performed a retrospective study of hip fractures in geriatric trauma patients. Hip fracture patterns were based on ICD -9 CM diagnostic codes for hip fractures (820.00-820.9). Patient variables were patient demographics, mechanism of injury, injury severity score, hospital and ICU length of stay, co-morbidities, injury location, discharge disposition, and in-patient mortality.

**Results:**

A total of 325 patient records met the inclusion criteria. The mean age of the patients was 82.2 years, and the majority of the patients were white (94%) and female (70%). Hip fractures patterns were categorized as two fracture classes and three fracture types. We observed a difference in the proportion of males to females within each fracture class (Femoral neck fractures Z-score = -8.86, p < 0.001, trochanteric fractures Z-score = -5.63, p < 0.001). Hip fractures were fixed based on fracture pattern and patient characteristics. Hip fracture class or fracture type did not predict short-term outcomes such as in-hospital or ICU length of stay, death, or patient discharge disposition. The majority of patients (73%) were injured at home. However, 84% of the patients were discharged to skilled nursing facility, rehabilitation, or long-term care while only 16% were discharged home. There was no evidence of significant association between fracture pattern, injury severity score, diabetes mellitus, hypertension or dementia.

**Conclusions:**

Hip fracture patterns differ between geriatric male and female trauma patients. However, there was no significant association between fracture patterns and short-term patient outcomes. Further studies are planned to investigate the effect of fracture pattern and long-term outcomes including 90-day mortality, return to previous levels of activity, and other quality of life measures.

## Introduction

Geriatric trauma in the US is on the rise and at our level-I trauma center we have seen a dramatic increase in our "G-60" geriatric trauma mechanism of injury. There has also been a shift from motor vehicle collision to falls as the new number one mechanism of injury in geriatric trauma. Annually in the US, there are over 300,000 hospital admissions due to hip fractures in geriatric patients. [1] Patients who sustain hip fractures are exposed to significant morbidity [1] and high mortality [2] at a treatment cost between 10.3 to 15.2 billion dollars per year in the US [3, 4]. These observations illustrate that hip fractures, especially in the elderly, represent significant health and economic challenges in need of focused attention. Thus, trauma centers across the country are trying to develop ways to improve the quality of care given to elderly trauma patients, which includes a better understanding of hip fracture patterns.

The National Hip Fracture Database (NHFD) the largest UK-based national hip fracture audit with 180 contributing hospitals in England, Wales and Northern Ireland has reported differences in outcomes among patients with hip fractures [5]. Similar large observational studies from the US [6] have recently been published which underscore the importance of anesthesia technique on mortality and length of stay among patients who underwent surgery for hip fractures.

The term "hip fracture" most commonly refers to fractures of the proximal femur and are generally categorized as (a) femoral neck fractures and (b) trochanteric fractures including intertrochanteric fractures, greater trochanteric fractures and subtrochanteric fractures, and combined inter- and subtrochanteric fractures. Most epidemiologic studies consider only 2 categories, femoral neck and intertrochanteric. Although there are reports showing some decline in the incidence of femur fractures among patients who stopped smoking and drinking alcohol or have been treated for osteoporosis with vitamin D [7–9], mortality and morbidity due to hip fracture remains rather high [7, 9, 10]. Thus, models have been developed to predict hip fracture mortality [11, 12]. However, it remains unclear whether there is a difference in femur fracture pattern distribution (femoral neck vs. trochanteric) in geriatric trauma patients. It is also unknown if there is any association between geriatric G60 trauma patients’ fracture pattern and outcomes. The purpose of this study was to determine if there is a difference in patient characteristics or patient outcomes as a function of fracture patterns in geriatric trauma patients age 60 years and older.

## Methods

This retrospective study was reviewed and approved by Western Institutional Review Board. Patients were identified by query of the institutional trauma registry at our American College of Surgeons (ACS) verified level-I trauma center for all hip fractures by ICD 9 CM code 820–820.9. Patients with no femur fracture, those with acetabular fracture, or penetrating injuries were excluded. The study period was from August 2012 to February 2014.

**Figure 1:**
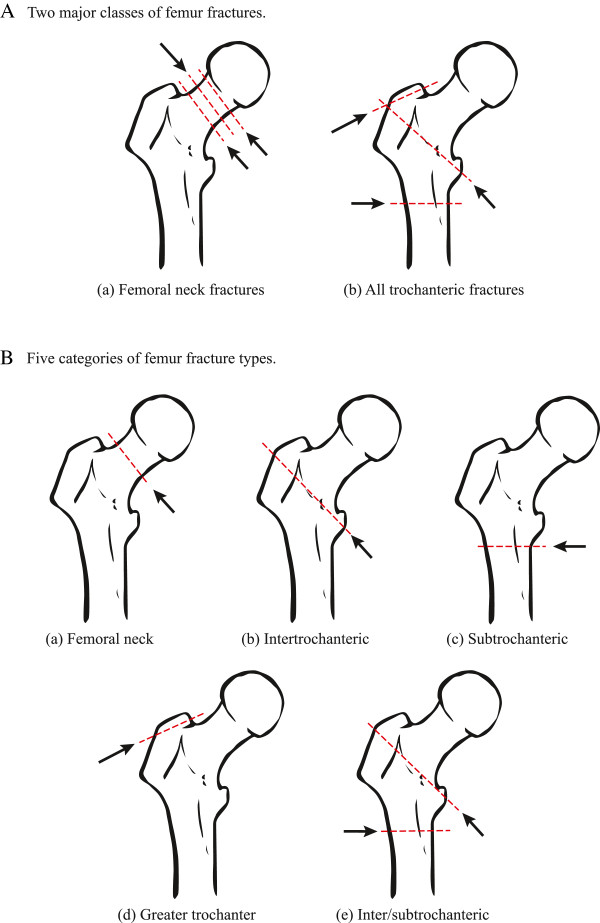
**Major classes of femur fractures (A) and categories of femur fracture types (B). A**. Femur fracture classes: (a) Femoral neck and (b) All trochanteric fractures. Red lines indicate fracture locations, as indicated by arrows. **B**. Femur Fracture Patterns: (a) Femoral neck (b) Intertrochanteric (c) Subtrochanteric (d) Greater trochanter (e) Combined inter & subtrochanteric. Red lines indicate fracture location, as indicated by arrows. For purposes of statistical analysis, patients with fractures corresponding to figures (c), (d), and (e) were grouped as "other". However, such grouping may not represent homogenous clinical category.

Data were collected both from the trauma registry and the electronic medical record. Data recorded included patient variables: age, gender, race/ethnicity, mechanism of injury, hospital and ICU length of stay, discharge disposition and mortality. Data captured were entered into Excel spreadsheets and an Access database. A trauma surgeon reviewed X-ray reports for each femur fracture, and if any ambiguity remained, a radiologist reviewed them. All fractures were described according to fracture class: (a) Femoral neck and (b) trochanteric fractures (Figure 1A). These classes were further categorized into following fracture types: (a) femoral neck, (b) intertrochanteric, (c) subtrochanteric, (d) greater trochanter, and (e) combined inter & subtrochanteric (Figure 1B). Trochanteric fracture class was a combination of intertrochanteric, trochanteric, and subtrochanteric fractures. Patient outcomes variables reviewed included: (a) hospital length of stay (HLOS) days, (b) ICU days (ICU LOS), and (c) discharge disposition including in-patient mortality. We examined the data for evidence of association between femur fracture type or fracture class and specific outcomes of interest. Covariates were age, gender, ethnicity/race, and injury severity score.

### Statistical analysis

Continuous variables were reported as means ± SD and categorical variables as percentages. Comparisons between groups were performed using analysis of variance (ANOVA) or Student’s t-test for continuous variables. Pearson’s Chi-square test, Fisher’s exact test, or two-proportion Z-test was used for categorical variables. Two-sided p-values were used and p < 0.05 was considered statistically significant.

## Results

### Demographics

There were 325 patients, who met all of the study inclusion criteria and none of the exclusion criteria. The mean age (years) was 82.2 ± 9.3 with range of 60 to 101 years. There were 70.2% (n = 228) female and 29.8% (n = 97) male patients. Falls accounted for 95% of mechanism of injury for these patients. Females were older (83.3 ± 8.8 years) than males (79.5 ± 9.8 years) on average (p = 0.001). The majority of subjects (94.2%, n = 306) were white, and all other ethnic groups composed the remaining 5.8% (n = 19) of the patient population. In reviewing patients’ co-morbidities, 191 (58.8%) patients had hypertension, 65 (20%) patients had diabetes mellitus, 51 (16%) patients had dementia, 49 (15%) patients had respiratory disease, 20 (6.2%) patients had chronic heart failure, and 20 (6.2%) patients were current smokers. We also studied the fall locations and discharge dispositions of the patients. There were 239 patients (73.5%) who fell at home, 18 patients (5.5%) who fell at nursing home, and 68 patients (21%) who fell at other locations. There were 40 patients (12.4%) who were discharged to home, 265 (81.4%) patients who were discharged to SNF, rehabilitation centers, or acute care centers, and 20 patients (6.2%) who either died or were discharged to hospice. The majority of patients fell from home, but the majority of patients were discharged to SNF, rehabilitation center, or acute care facility as it is shown in Table 1.

**Table 1:** **Description of discharge disposition in relation to their injury locations**

Injury locations	Discharge disposition	N	Percent
Home	Home	25	7.7%
	SNF/Rehab/LTC	200	61.7%
	Hospice/Died	13	4.0%
Nursing home	Home	4	1.2%
	SNF/Rehab/LTC	12	3.7%
	Hospice/Died	2	0.6%
Other	Home	11	3.4%
	SNF/Rehab/LTC	52	16.1%
	Hospice/Died	5	1.5%

SNF/Rehab/LTC represents skilled nursing facility, rehabilitation, and long term care. This table represents demographic description of the patients’ discharge disposition in relation to their injury locations. There was no statistical analysis performed on this data due to low numbers in some of the sub-groups.

### Fracture class (femoral neck and trochanteric fractures) among elderly G-60 patients

**Figure 2:**
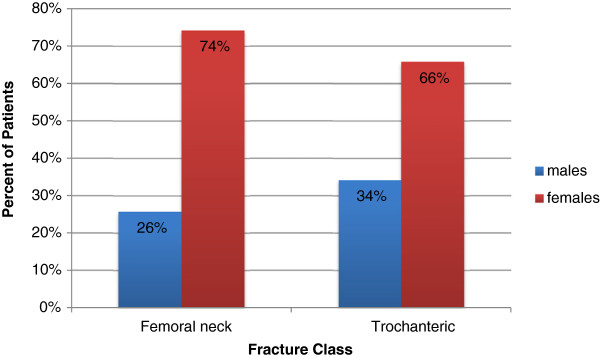
**A comparison of proportions of males and females for femoral neck and trochanteric fractures.** Femoral neck fractures for males is 43/97 and for females 124/228. Trochanteric fractures for males is 54/97 and for females 104/228, Z-score = -5.63, p < 0.001.

Among our study subjects, 51.4% (n = 167) had femoral neck fractures, and 48.6% (n = 158) had trochanteric fractures. The mean (SD) age of patients with femoral neck fracture was 82.7 ± 8.5 years, and the average age of patients with trochanteric fractures was 82.5 ± 9.6 years (p = 0.84). The difference in age was not statistically significant, (251df) = -0.149, p = 0.881. Patients’ mean (SD) injury severity scores (ISS) for femoral neck and trochanteric fractures were 9.4 ± 2.0 and 9.8 ± 3.1, respectively (p = 0.20). We also determined the distribution of femoral neck and trochanteric fractures among the patients based on their gender, and the proportion of males and females with trochanteric fractures and femoral neck fractures showed significantl differences as shown in Figure 2. The HLOS and ICU LOS for each fracture class were also examined. The patients with femoral neck fractures stayed on average (SD) 5.1 ± 2.6 days in hospital, and the patients with trochanteric femur fractures stayed on average 5.4 ± 2.7 days (p = 0.341). The ICU LOS for patients with femoral neck and trochanteric fractures were on average (SD) 3.7 ± 3.6 and 3.3 ± 2.2 days (p = 0.662), respectively. The fall locations and the discharge dispositions were also studied and compared between two fracture classes, and the distribution of each fracture class was similar in various locations. Table 2.

**Table 2:** **Fracture class, fall locations and discharge dispositions**

		Femoral neck (N, %)	Trochanteric (N, %)
Fall locations	Home	124 (74.3)	115 (72.8)
	Nursing home	9 (5.4)	9 (5.7)
	Other	34 (20.4)	34 (21.5)
	Total	167 (100)	158 (100)
Discharge disposition	Home	21 (12.6)	19 (12.1)
	SNF/Rehab/LTC	138 (82.6)	126 (80.3)
	Died/hospice	8 (4.8)	12 (7.6)
	Total	167 (100)	158 (100)

SNF/Rehab/LTC represents skilled nursing facility, rehabilitation and long-term care. Pearson’s chi-square (2 df) = 0.09, p = 0.979 for fall locations. Pearson’s chi-square (2 df) = 1.14, p = 0.566 for discharge disposition.

### Femoral fracture types in elderly G-60 patients

**Figure 3:**
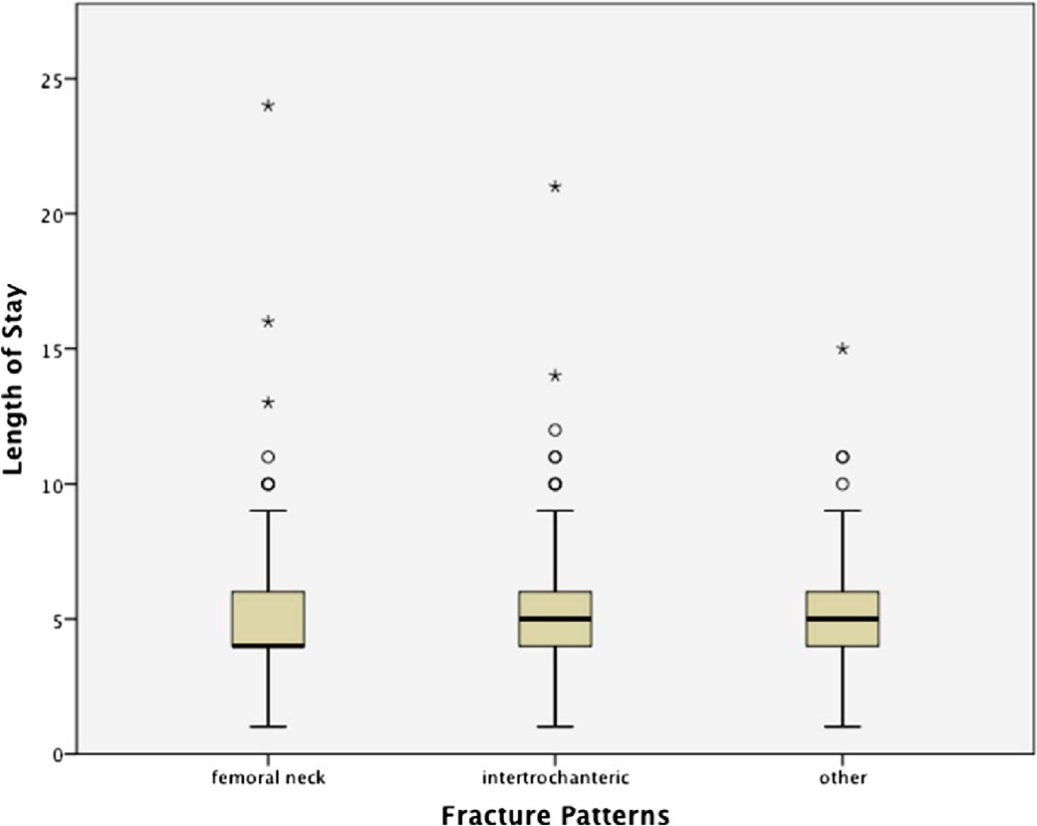
**Box plot of HLOS and femur fracture patterns: Femoral Neck, Intertrochanteric, and Other.** Results show variations in hospital length of stay among different types of fracture patterns. Using a one-way analysis of variance (ANOVA), there was not a significant effect of fracture patterns on hospital length of stay among the three fracture patterns (femoral neck, intertrochanteric, and other). F (2, 321) = 0.841, p = 0.432.

When the patients’ fracture types were compared, there were 167 patients (51.4%) with femoral neck fractures, and 129 patients (39.7%) with intertrochanteric fractures. The remaining 29 patients (8.9%) had subtrochanteric, greater trochanter only, or combined inter- and subtrochanteric fractures, and were considered as "other" for the purpose of meaningful statistical analysis. The fracture types for females, males, and both sexes were explored, and are shown in Table 3. There were higher proportions of femoral neck and intertrochanteric fractures in females compared to males. The average age was the highest in patients with intertrochanteric fractures (83.1 ± 9.4 years). The average age of patients who had femoral neck fracture was 82.1 ± 8.7 years, and other fracture was 78.5 ± 10.9 years (p = 0.044). The HLOS for each fracture type was analyzed using one-way analysis of variance (ANOVA), and the average HLOS were similar across the fracture types as shown in Figure 3. There were total of 35 ICU admissions among the patient population. Some of the patients with hip fracture were admitted to ICU due to following reasons including, but not limited to: patients with or on anticoagulation (e.g. Coumadin), significant cardiac history (e.g. pace-makers), hemodynamic instability, arrhythmia, confusion, concussion, mechanical ventilation, or renal dialysis. The average ICU days for patients with femoral neck fractures were 3.7 ± 3.6 days (n = 15), intertrochanteric fractures were 2.8 ± 1.9 days (n = 16), and other fractures were 5.5 ± 2.4 days (n = 4).

**Table 3:** **Hip fracture type distribution for males and females**

Fracture types		Gender		Total
		Female	Male	
Femoral neck	Count (N)	124	43	167
	% within fracture pattern	74.3%	25.7%	100.0%
	% within gender	54.4%	44.3%	51.4%
Intertrochanteric	Count (N)	87	42	129
	% within fracture pattern	67.4%	32.6%	100.0%
	% within gender	38.2%	43.3%	39.7%
Other	Count (N)	17	12	29
	% within fracture pattern	58.6%	41.4%	100.0%
	% within gender	7.5%	12.4%	8.9%
Total	Count (N)	228	97	325
	% within fracture pattern	70.2%	29.8%	100.0%
	% within gender	100.0%	100.0%	100.0%

The table shows the number of patients (n) and associated percentage (%) for gender and hip fracture types. Pearson’s Chi-Square (2df) = 3.63, p = 0.162.

#### Discharge disposition

We completed analysis of the effect of femur fracture class on discharge disposition as shown in Table 2. There were no significant associations between fracture class and discharge disposition, (2 df) = 1.14, p = 0.566.

#### The effect of age on fracture type

**Figure 4:**
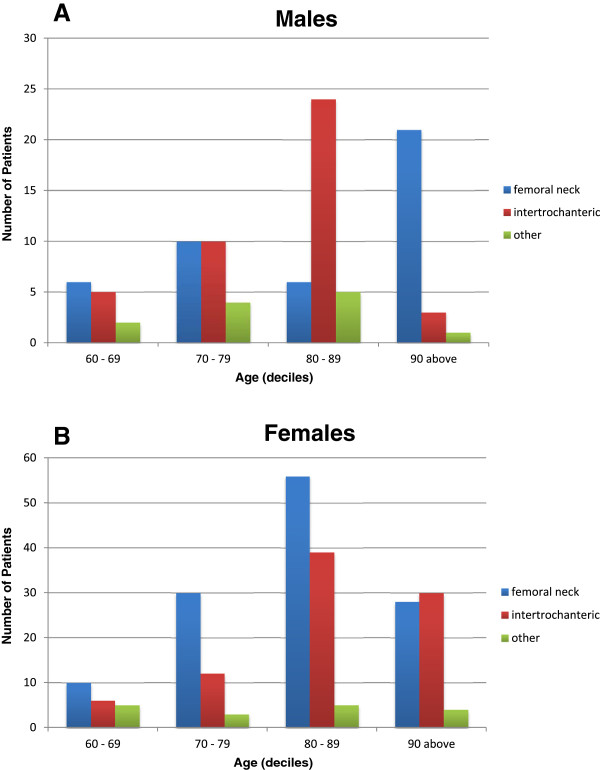
**Femur fracture patterns and age in deciles for male (A) and female (B) patients. A**. Age group (decile years) and femoral fracture patterns in males. The frequency of different fracture patterns (Femoral neck, Intertrochanteric, and Other) in adult males (decile years) aged 60 and above. Pearson’s Chi-square (6 df) = 27.6, p < 0.0001. **B**: Age group (decile years) and femoral fracture patterns in females. The frequency of different fracture patterns (Femoral neck, Intertrochanteric, and Other) in adult females (decile years) aged 60 and above. Pearson’s chi-square (6 df) = 14.5, p = 0.023.

We examined the relationship between patient age in deciles and femoral fracture type in males (Figure 4A) and females (Figure 4B). Males had the highest number of patients with femoral neck fracture in "90 and above" age group and the highest number of patients with intertrochanteric fractures in "80-89" age group. On the other hand, females had the highest number of patients with both femoral neck and intertrochanteric fractures in "80-89" age group. There were significant differences between the patient age in deciles and femoral fracture type in males (p < 0.0001) and females (p = 0.023). We also performed an Analysis of variance (ANOVA) using patient age as continuous variables. The result showed that hip fracture types used as categorical variables were not associated with patient age as a continuous variable, F (4, 248) = 0.600, p = 0.66.

#### Surgical treatment

Hip fractures were fixed by orthopedic surgeons based on standard of care. Among the 325 patients with hip fractures 25 had total hip replacement, 80 had partial hip replacement, 9 had internal fixation without reduction, 207 had open reduction with internal fixation and 2 patients had closed reduction of dislocation of the hip.

## Discussion

The primary findings of this study described femur fracture patterns in a group of elderly trauma patients with hip fractures. The study group was predominantly white (94%), female (70%) and in their 8th decade of life. As reported in previous studies [13] and confirmed in this study, about half (51.4%) of the study subjects were diagnosed with femoral neck fractures, whereas 48.6% had trochanteric fractures. Patients in the two fracture groups did not differ in age or injury severity scores. The prevalence of femoral neck fractures was higher in females than in males. Similarly, The prevalence of trochanteric fractures was higher in females than in males. The ratio of male to female in femoral neck fracture was 1:3 and the ratio of male to female in intertrochanteric fracture was 1:2. This is confirmatory of previous study done by Brunner et al. [14], although gender alone was not a significant predictive factor for femoral neck or trochanteric fractures (p = 0.097). The observed higher prevalence of femoral neck fractures in females was not explained by the older age of females. We concluded from our observations that the higher prevalence of femoral neck fractures may be a characteristic of white geriatric females in our service area.

The key question we raised in the introduction to this study was whether there were any connections between hip fracture pattern (class or type) and short-term inpatient outcomes. As reported in the results section, femoral neck or trochanter fractures classes had no effect on HLOS (p = 0.706), ICU-LOS (p = 0 .712), or patient discharge disposition including the number of inpatient deaths (Pearson Chi-Square (2df) = 0.313, p = 0.855). Similarly, fracture types had no effect on HLOS (p = 0.814).

In regards to the classification scheme, certainly AO/OTA classification provides more detail. However, use of this scheme would produce many subgroups, which would need to be grouped as we did for the purpose of analysis. Therefore, for the purpose of this study, we classified the fracture patterns in groups consistent with ICD-9 CM codes. This is also well described in other publications [7, 15, 16] as a method of categorizing the different types of hip fractures.

There are several factors that influence HLOS and complications such as surgical technique and whether arthroplasty was cemented or uncemented. The majority of hip fracture patients in our study (207/325) or 64% underwent open reduction with internal fixation. The influence of this group relative to patients who underwent total hip replacement (7.7%) internal fixation without reduction (2.7%), closed reduction of dislocation of hip (0.6%) on outcomes, and partial hip replacement (24.8%) needs to be taken into account when interpreting outcomes. We also suspect a higher proportion of patients with hip fractures were admitted to the ICU at our level-I trauma center than would be the case in UK and Europe.

### Study limitations

This was a retrospective chart review limited to a single level-I trauma center.

## Conclusion

Our examination of fracture pattern in geriatric trauma patients showed no association between fracture patterns and outcomes including hospital length of stay, ICU length of stay, and discharge disposition. Males and females did differ in fracture patterns, but these differences were not associated with different outcomes. Further studies should include greater diversity in demographics, and linkage of fracture patterns to outcomes such as pain scores, infection rate, hospital readmission and patient experience. As elderly population continues to rise and trauma centers are seeing more elderly patients with fall and associated hip fractures, we must continue to explore ways to decrease mortality and morbidity related to hip fractures.

## Abbreviations

ICD-9 CM: International Classification of Diseases, Ninth Revision, Clinical Modification
ISS: Injury severity score
ICU: Intensive Care Unit
G-60: Geriatric trauma service
LOS: Length of stay
SNF: Skilled nursing facility
Rehab: Rehabilitation
LTC: Long term care.
